# Lifespan Differences in Hematopoietic Stem Cells are Due to Imperfect Repair and Unstable Mean-Reversion

**DOI:** 10.1371/journal.pcbi.1003006

**Published:** 2013-04-18

**Authors:** Hans B Sieburg, Giulio Cattarossi, Christa E. Muller-Sieburg

**Affiliations:** Stem Cell and Regenerative Medicine Program, The Sanford-Burnham Medical Research Institute, La Jolla, California, United States of America; University of Notre Dame, United States of America

## Abstract

The life-long supply of blood cells depends on the long-term function of hematopoietic stem cells (HSCs). HSCs are functionally defined by their multi-potency and self-renewal capacity. Because of their self-renewal capacity, HSCs were thought to have indefinite lifespans. However, there is increasing evidence that genetically identical HSCs differ in lifespan and that the lifespan of a HSC is predetermined and HSC-intrinsic. Lifespan is here defined as the time a HSC gives rise to all mature blood cells. This raises the intriguing question: what controls the lifespan of HSCs within the same animal, exposed to the same environment? We present here a new model based on reliability theory to account for the diversity of lifespans of HSCs. Using clonal repopulation experiments and computational-mathematical modeling, we tested how small-scale, molecular level, failures are dissipated at the HSC population level. We found that the best fit of the experimental data is provided by a model, where the repopulation failure kinetics of each HSC are largely anti-persistent, or mean-reverting, processes. Thus, failure rates repeatedly increase during population-wide division events and are counteracted and decreased by repair processes. In the long-run, a crossover from anti-persistent to persistent behavior occurs. The cross-over is due to a slow increase in the mean failure rate of self-renewal and leads to rapid clonal extinction. This suggests that the repair capacity of HSCs is self-limiting. Furthermore, we show that the lifespan of each HSC depends on the amplitudes and frequencies of fluctuations in the failure rate kinetics. Shorter and longer lived HSCs differ significantly in their pre-programmed ability to dissipate perturbations. A likely interpretation of these findings is that the lifespan of HSCs is determined by preprogrammed differences in repair capacity.

## Author Summary

All hematopoietic stem cells (HSCs) are characterized by the capacities to produce all blood cell-types by differentiation and to maintain their own population through self-renewal divisions. Every individual HSC, therefore, can generate a complete blood system, or clone, conveying oxygenation and immune protection for a limited time. The time for which all mature blood cell-types can be found in a clone is called the lifespan. Interestingly, HSCs with different lifespans co-exist in the same host. We address the unresolved question: what controls the lifespan of HSCs of the same genotype exposed to the same environment? Here, we use a new approach to multi-scale modeling based on reliability theory and non-linear dynamics to address this question. Large-scale fluctuations in the experimental failure rate kinetics of HSC clones are identified to predict small-scale, genome level, events of deep penetrance, or magnitudes that approach population size. We broadly find that one condition explains our experimental data: repair mechanisms are a priori imperfect and do not improve, nor deteriorate, during the lifespan. As a result, progressively “worse-than-old” genome replicates are generated in self-renewal. A likely interpretation of our findings is that the lifespan of adult HSCs is determined by epigenetically pre-programmed differences in repair capacity.

## Introduction

Adult tissue stem cells, such as hematopoietic stem cells (HSCs), are distinguished from mature cells by the ability to generate all mature cell-types of a particular tissue (multi-potency). To generate mature cells, HSCs differentiate into cells of lower potency. The resulting loss of stem cells must be compensated for by self-renewal, i.e. cell divisions which preserve the multi-potential differentiation capacity of the ancestral HSC. The reliability with which HSCs can transfer their identity and maintain self-renewal upon proliferation has been of keen interest to the field [1], [2]. Important questions are: Are daughter HSCs “as good as old” after self-renewal? How often can individual HSCs self-renew? Do different HSCs have different self-renewal capacities? What controls the fidelity of self-renewal? These questions remain incompletely understood.

Because of their extensive self-renewal capacity, HSCs were initially thought to be immortal. This view was supported by the observation that populations of HSCs could be serially transplanted for a very long period of time - exceeding the normal lifespan of the donor [3], [4]. However, when HSCs were examined on the clonal level, extensive heterogeneity in lifespan was revealed [5]–[7]. A detailed analysis of a large panel of HSCs showed that the lifespan of individual HSCs is mathematically predictable [8]. HSCs with lifespans from 10 to nearly 60 months were found side-by-side in the same donor [8], indicating that the lifespan is pre-determined on the level of each HSC. Because lifespans of single transplanted HSCs are predictable from few initial values of their repopulation kinetic, the lifespan is a programmed HSC-specific property [8]. The population dynamics, therefore, predict that the molecular machinery which preserves self-renewal, will ultimately fail.

Several hypotheses have been developed to identify and explain how HSCs limit their lifespan. The generation-age hypothesis [9] states that for every cell division, an HSC loses some quality that is referred to as “stemness”.

According to Hayflick's hypothesis [10], the probability that somatic cells produce viable daughter cells which can themselves divide, decreases as the number of divisions increases. The decrease might be caused by progressive telomere shortening [10]. Hence, an extension of Hayflick's hypothesis predicts that stem cell self-renewal capacity should be self-limiting at the level of individual HSCs.

Yet, HSCs and other stem cells, express telomerase [11]–[14]. This enzyme repairs telomere damage and, thus, aids in preserving genomic integrity. Thus, telomere shortening alone is unlikely to explain a limited lifespan of HSCs. Indeed, mice that have been homozygously ablated for telomerase activity show only mild effects and need to be severely stressed to reveal deficiencies in the hematopoietic system [15]. Potentially in line with these findings in mice are clinical data. It was suggested that telomerase expression declines in the long-run and may be a cause for late bone marrow transplant failure [16]. Declining telomerase expression may act in conjunction with the high stressor load imposed by the many co-morbidities affecting transplant patients [17].

Another proposal suggested that, in conjunction with oxidative stresses, high levels of reactive oxygen species (ROS) could be a damaging force acting on the long-term repopulating capacity of HSCs [18], [19]. The corresponding restoring force is provided by Forkhead box class O (FoxO) transcription factors. FoxO transcription factors increase the expression of genes whose products blunt the effects of elevated ROS [20]–[22]. That different sources of self-renewal failures could be causally co-dependent is suggested by findings that oxidative stress could shorten telomeres [23].

Along-side genome stability, the preservation of epigenetic patterning is an important prerequisite to reliably produce functional daughter HSCs upon self-renewal. It has been suggested that both maintenance and de novo methylation are needed to maintain epigenetic stability [24]. The expression levels of DNA methyltransferases [25], [26] responsible for maintenance (DNMT1) and de novo methylation (DNMT3a and DNMT3b) could be important for restoring HSC multi-potency [27], [28]. Quantitative work has suggested that small failures may accumulate over time in the DNMT1 pathways leading to the loss of maintenance methylation and, ultimately, epigenetic stability [29]. Yet, neither of these mechanisms and hypotheses explain how HSCs with different lifespans co-exist in a single host.

It was suggested that HSCs could preserve their functional integrity over long periods of time by alternating between two states, called resting or quiescent, and active, respectively [30]–[32]. This idea poses that intermittent transitions to quiescence could provide the time needed to minimize the detrimental effects of repeated DNA replication and other stresses on the HSC population as a whole [33]. Elegant mathematical models of this idea have been formulated [34]–[36]. Surprisingly, quiescence may leave HSCs more vulnerable to mutations following DNA repair [37]. Quiescent and active HSCs may use different DNA repair mechanisms and the restoring pathway used by quiescent HSCs may lead to higher differentiation probabilities following re-activation. Never-the-less, when HSC quiescence was inhibited by the expression of the Wnt inhibitor Dickkopf-1, the HSC pool exhausted prematurely [38]. This suggests that periods of rest in the niche are essential for controlling HSC lifespan - supporting the idea that repair is necessary to maintain HSC lifespan.

**Figure 1:**
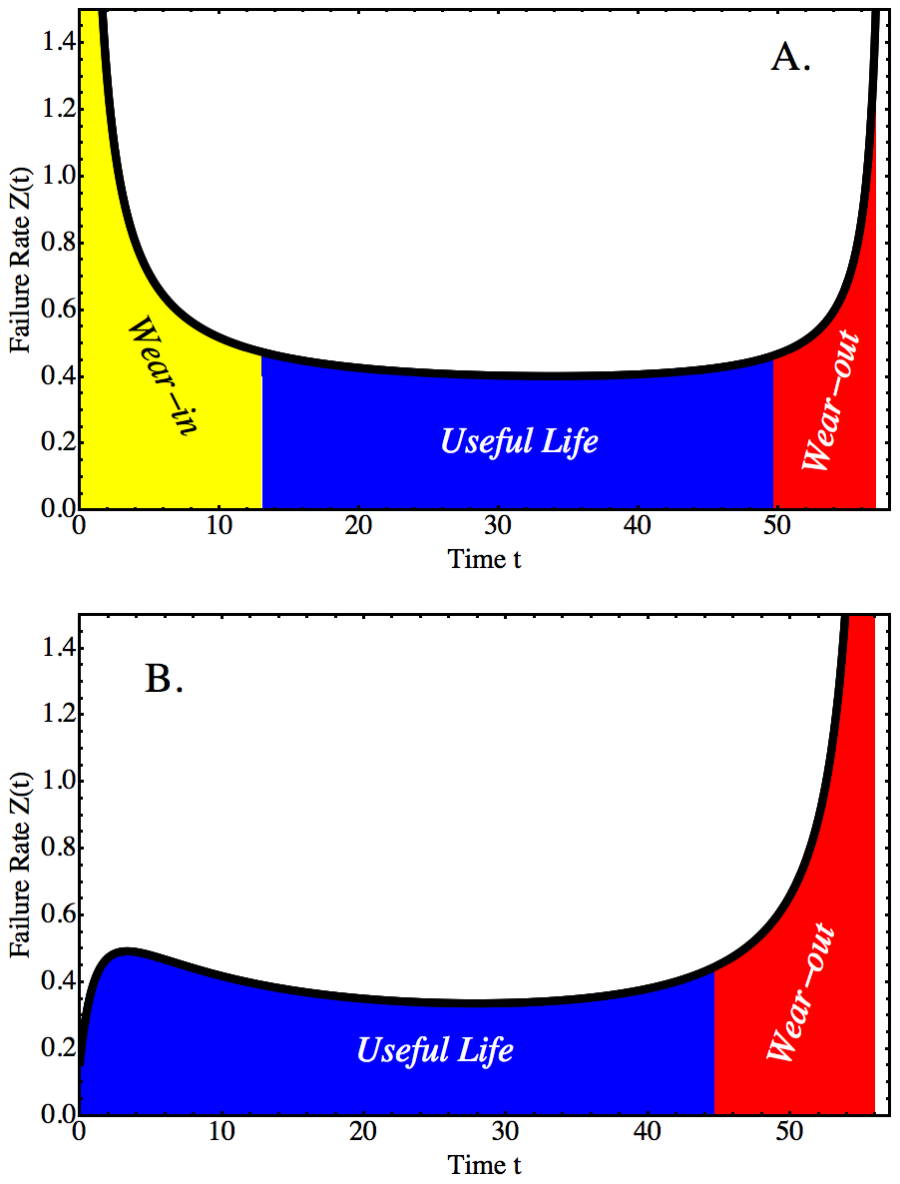
Failure Rate Kinetics of Machine and Clonal Blood Cell Populations. Schematic representations of the failure rate kinetics (vertical axis) of two systems over time (horizontal axis): A. Population of machines; B. Population of cells derived from a single long-term repopulating hematopoietic stem cell (HSC). In reliability theory, it is thought that three major phases describe the shape of the failure rate function (the black curve in Parts A and B was generated for demonstration purposes using appropriate mathematical functions). A: For populations of machines, the “bathtub” shape is thought to begin with a “wear-in” phase (yellow). During “wear-in”, factory defective items are flushed out. The population, there-after, reaches the so-called “useful life” period (blue), where failure rates are minimized. The “bathtub” is completed by the third phase of “wear-out” (red), where many essential parts fail in an increasingly larger number of machines. B: The biology of clonal stem cell populations may lead to a different assembly of phases, generating a different shape of the failure rate curve. Unlike for machines, the clonal population creates itself during expansion. Consequently, a direct analog of “wear-in” may not exist, or may be short, and not characterized by failure rate decrease. The “useful life” period may, therefore, extend to the start of the failure rate curve. “Wear-out” may occur for reasons similar to machine populations, i.e. through the accumulation of failures in an increasingly larger number of HSCs. The present paper uses an interdisciplinary approach combining the analysis of experimental data, mathematical reasoning and computer simulation to determine the actual shape of clonal failure rates and make predictions about the dynamical mechanisms responsible for failure accumulation and clonal extinction. The goal of this approach is to find new experimentally testable hypotheses about how stem cells autonomously control their growth using “built-in” failure as a passive mechanism against cancerous proliferation.

Mathematically, the lifespan of populations has been addressed in manufacturing, engineering, actuarial and biological applications of reliability theory [39]. Reliability theory was first developed as a quality control tool to predict the time-to-failure - the manufacturing term for lifespan - of manufactured goods to determine warranty times. When examined as a population, the lifespan of manufactured goods proceeds through well-defined phases (Figure 1). First, a decline in population size is found, which is interpreted as failure due to factory error. Second, there is a period of little change, known as the useful phase of the population of goods. Thereafter, the population size declines again, this time caused by age-related failure of essential machine components (wear-out phase). The second phase can be prolonged, if goods are repaired. If repairs occur repeatedly, the useful life will be extended, yet the population of goods will fail in the end, because of a general deterioration of many essential parts. In biology, failure theory has been applied to develop general laws of aging and longevity [40]–[42], respector-ligand dissociation [43], or genome instability [44]. Here, we show that the principles of reliability and failure can be exploited to craft a new model of HSC self-renewal suggesting that HSCs differ a priori in the number of (self-)repair cycles they can undergo.

## Results

### Repopulation Time-to-Failure

We obtained repopulation data experimentally by transplanting single HSCs into ablated mice as described previously [45]–[48]. The donor HSCs and the host type mice differed in the allelic forms of the Cluster of Differentiation 45 (CD45) antigen. The CD45 antigen is expressed in most hematopoietic cells. This allowed us to follow the mature progeny derived from the transplanted HSCs by staining white blood cells for the donor-type marker. Mice were analyzed every other month and the percent of white blood cells that stained for the donor-type marker were recorded (% donor-type cells). Together, all the data points form the repopulation kinetic as a time series (compare Algorithm 1, Input specification). Because all cell populations were derived from a single HSC, the repopulation kinetic of the clone represents the total repopulation capacity of the original HSC.

**Algorithm 1. **Algorithm for calculating the discrete time series of reliabilities failure probabilities failure densities and failure rates for batches of raw kinetics. Numbers to the left of the listing indicate line numbers. These are used to reference particular computations in the main narrative. The general notation 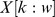 denotes the elements 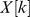 through 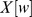 of a list 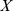. The symbol 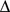 denotes the successive differences operator defined for a list of length 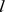 by 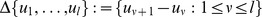. The function Sum(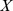) sums the elements of a list 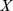.

**input** : A batch of 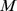 kinetics 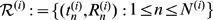 of lengths 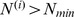, 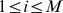. By 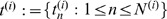 with 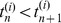 we denote the 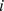-th time scale, and by 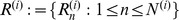 the 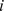-th kinetic measured experimentally. 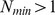 is a minimum lower bound on time series size needed to conduct meaningful analyses.

**output**: A list of lists 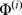 of time series: Time scale (symbolically: 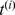), Process Rate (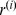), Reliability (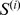), Failure Probability (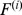), Failure Density (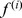), Failure Rate (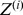), for 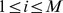.

**1 for** 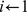 **to** 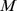 **do**

**2** *process the* 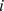 *-th repopulation kinetic* 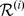;

**3** 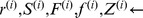 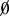; **// clear all lists;**

**4** 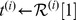; **// extract time scale;**

**5** 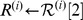; // **extract process measurements**;

**6** 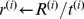; **//determine instantaneous rates;**

**7** 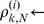 Sum 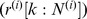;

**8** 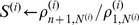; **// reliability;**

**9** 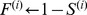; **// failure probability**;

**10** 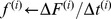; **// failure density**

**11** 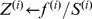; **// failure rate**;

**12** 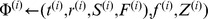; **// reliability profile of** 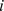 **-th kinetic;**

**Figure 2:**
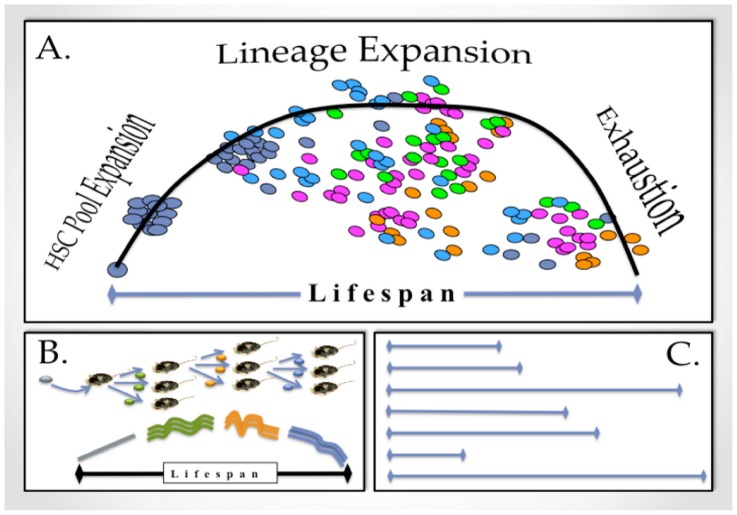
The Life of A Hematopoietic Stem Cell. A: Limited lifespan: When a monoclonal hematopoietic system is initiated by transplanting a single HSC (dark blue sphere), it expands to a pool of clonal HSCs through self-renewal (cluster of blue spheres). This pool distributes through the organism. HSCs differentiate to generate mature cells of all lineages (shown as magenta, orange, green, light-blue spheres). This process depends on the intrinsic properties of the founder HSC [63], [65]. The overall output of mature cells in blood (measured in %-donor type cells; vertical axis (not shown in the figure)) over time (horizontal axis labelled “Lifespan”) is indicated by the black curve. For all normal HSCs, this kinetic has a ballistic shape, thus indicating that a clone's ability to produce mature cells of all major lineages (the lifespan) is limited. The lifespan is mathematically predictable with high accuracy from few initial points of the repopulation kinetic [8]. B: Programmed Lifespan: When daughter HSCs derived from a single ancestral HSC are transplanted into separate hosts, the repopulation kinetics are very similar (modified from [2]). In particular, all daughter HSCs become extinct at the same time [8]. This suggested that the lifespan is epigenetically fixed (programmed) and heritable in self-renewal. C: Lifespan Diversity: The relialogram illustrates that when HSCs are sampled from bone marrow, lifespans of different durations are found [2], [66], [67]. Therefore, the length of time for which HSCs can repopulate an ablated host varies according to the epigenetic programs of individual HSCs.

We previously showed that any HSC will eventually fail to repopulate all mature cell populations [8] (also compare summary Figure 2). The time period until the multi-potential repopulation capacity fails was called the HSC lifespan. Since the lifespan marks the loss of multi-potency, it is the time-to-failure of a stem cell clone as a system [39]. We showed previously that the time-to-failure of individual HSCs is mathematically predictable with great accuracy from few initial measurements of the repopulation kinetic [8]. Hence, the time-to-failure (lifespan) is a deterministic property of individual HSCs, but can be treated as a (Gumbel-distributed) random variable at the level of the HSC compartment.

Statistical analysis of 38 repopulation kinetics ascertains that the time-to-failure estimator is unbiased and almost efficient (compare Text S1). This means that the repopulation kinetic of a clone provides near optimal information about the time-to-failure. Of note, the time-to-failure can be modeled as a power law of the proliferative capacity of the clone (compare Text S1, eq 1). From the traditional interpretation of a power law relationship (for example, see [49]), we can expect that reliability and failure rate analyses of the clonal time-to-failure are unaffected by the time scale on which repopulation data were obtained. Therefore, the systems reliability approach can be applied to repopulation kinetics obtained using different time scales (for example, [50]).

### Repopulation Reliability

The time-to-failure 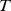, or lifespan, of an HSC, is a deterministic quantity that measures the time until at least one mature cell population is no longer repopulated. 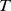, therefore, equals the time to the first, and last, failure of multi-potency in all, not just single, clonal HSCs.

In systems theory, reliability is defined as a conditional probability ([39]; also compare Materials & Methods (M&M), section “Reliability Theory”). Specifically, a system is said to operate reliably, if its strength is likely to exceed its load at a future time, given that the system has operated within specifications up to the present time.

The strength of a clonal system lies in the self-renewal and multi-potential differentiation capacities of its HSC population. Here, the term “capacity” can be given the rigorous quantitative meaning of “obtainable work”. Clonal experiments measure the amount of “realized work”, i.e. how much of the strength has actually been transformed into new HSCs (by self-renewal) and mature cells (by differentiation) over time, given load.

**Figure 3:**
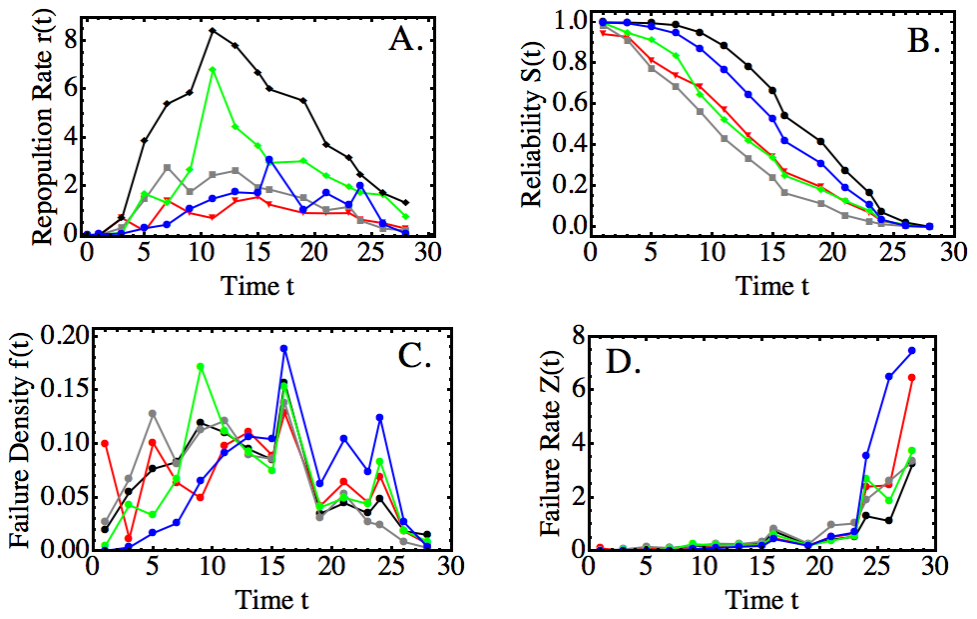
Reliability and Failure Kinetics of a Long-lived Long-term Repopulating HSC. A–D: Four types of kinetics were calculated (compare Algorithm 1) from experimental kinetics for all clonal cell populations together (black), and the myeloid (green), T lymphocyte (red), and B lymphocyte (gray) cell populations, separately. In the representative example shown, notation is as in Algorithm 1 (applied to a single kinetic, i.e. batch size 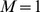). Also shown are the respective kinetics for the population of clonal hematopoietic stem cells (HSCs; blue). Since population data are difficult to obtain for stem cells directly, the HSC-related kinetics were inferred from the other data. This was accomplished by first predicting the reliability (Part B, blue curve) using the structure balance eq 1 and, then, deriving the other kinetics (blue curves for C, D, then A) with the methods of Algorithm 1.

Therefore, we could use the repopulation kinetics to identify the rate at which “work” is performed to quantitate clonal reliability. Specifically, we defined the clonal repopulation reliability as the normalized area under the curve of the repopulation rate kinetic (compare Algorithm 1, Line 8; also see Figure 3, A and B). Of note, the clonal reliability can be estimated even if only few initial repopulation data are known, since an HSC's lifespan is predictable from the first few measurements of its repopulation kinetic [8].

Using the repopulation data of 38 HSC clones with lifespans ranging from 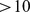 to nearly 60 months (compare Figure S1 in Text S1), we first determined the repopulation rate kinetics for the whole clone (parameters shown in Table S1 of Text S1) and also for all major subpopulations of T cells, B cells and myeloid cells (representative example shown in Figure 3, A; calculation: Algorithm 1, Line 6). Next, we used the clone data to calculate the respective reliabilities (Figure 3, B; calculation: Algorithm 1, Line 8). An additional curve can be inferred, if the clonal structure is considered in a so-called “common cause” model of clonal reliability. A common cause model poses that, in a multi-component system, the unknown reliability of a central component can be approximately determined by the relationship of all remaining system components to the system's reliability ([39], pp. 217–222).

Since all mature lineages derive from HSCs, a mature cell population and the HSC population can be viewed as serially connected system components. On the other hand, the populations of mature cells appear connected in parallel, since the failure of one such population does not imply the failure of all. The serial connectivity is a mathematical way of representing multi-potency. Hence, an inferred reliability structure model should generate the reliability kinetic of the clonal HSC population.

Considering the HSC population a “common cause” with reliability denoted 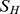, we posed that the clonal reliability (denoted 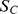) is connected to the reliabilities of the T cell, B cell and myeloid cell populations (denoted 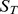, 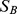, 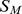, respectively) by:
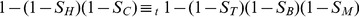
(1)
Eq 1 is a balance formula expressing the conservation of system structure over the life of a clone. It states that two parallel structure models of a clone behave similarly for all times 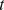 (indicated by the notation 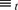). The first model (left-hand side, eq 1) considers the HSC population and the clone as a parallel reliability structure. The second model (right-hand side, eq 1) quantitates the reliability of the major mature cell-types as a parallel structure. We applied eq 1 to calculate the values 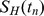 at discrete time points 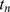 given by our data. The inferred kinetics are shown as blue curves in Figure 3.

The common cause argument suggests that the HSC population reliability closely resembles the reliability of the clone, modulo a time lag that is small compared to the lifespan (Figure 3, B). This outcome is consistent with our previous conclusions, obtained by different methods that the information contained in the repopulation time series predicts HSC behavior [8].

### Repopulation Failure Probability and Failure Density

By definition, the reliability is a forward looking measure that predicts the chances that a system will continue to operate according to its specifications for some time into the future, provided that it has operated reliably in the past. For HSCs, reliable operation means that their main characteristics, i.e. self-renewal and multi-potential differentiation capacities, are preserved when these cells divide. What are the chances of unreliable operation?

In systems theory, unreliability is defined by the conjugate probability of the reliability (for computation compare Algorithm 1, Line 9). Specifically, the probability that the system fails, or failure probability, equals 1 minus the probability that it can continue to operate as before. Hence, the repopulation failure probability, or repopulation “unreliability”, is a cumulative probability function [39] (high/low values indicate high/low likelihood of failure). Its graphical representation is an S-shaped curve whose shape is a horizontally flipped image of the corresponding reliability kinetic (Figure 3).

The failure probability gives rise to the failure density. The latter is a probability density in the usual sense and defined by the rate with which the failure probability changes over time (small values mean little change, high values mean lots of change). We determined the failure probabilities and densities for all clonal populations (Figure 3, C and D; Algorithm 1, Line 10). Because we could predict the reliability kinetics of the HSC population (previous section; also Figure 3, B), we could predict an approximate shape for the repopulation failure density of the HSC population, as well.

Application of the method of symbolic time series comparison in [51] shows that the relationship between the failure densities of the subpopulations of a clone changes over time (Figure 3 C). Specifically, the repopulation failure densities of the HSC population and some mature cell populations becomes more similar (increasingly converge to the same symbol sequence (data not shown)), as the lifespan is approached. By contrast, the densities lack similarity at the beginning of clonal life. To clarify this observation, we conducted a more detailed analysis of the long-range failure dynamics of HSC clones with different lifespans.

### Repopulation Failure Rate Kinetics

In systems theory, the failure rate provides information about how system failure occurs as a function of system load acting against system strength over time [39]. Since the future behavior of HSCs is largely pre-programmed [8], [46], the failure rate kinetic informs about how strength, or capacity, is transformed into new HSCs (by self-renewal) and mature cells (by differentiation) over time.

**Figure 4:**
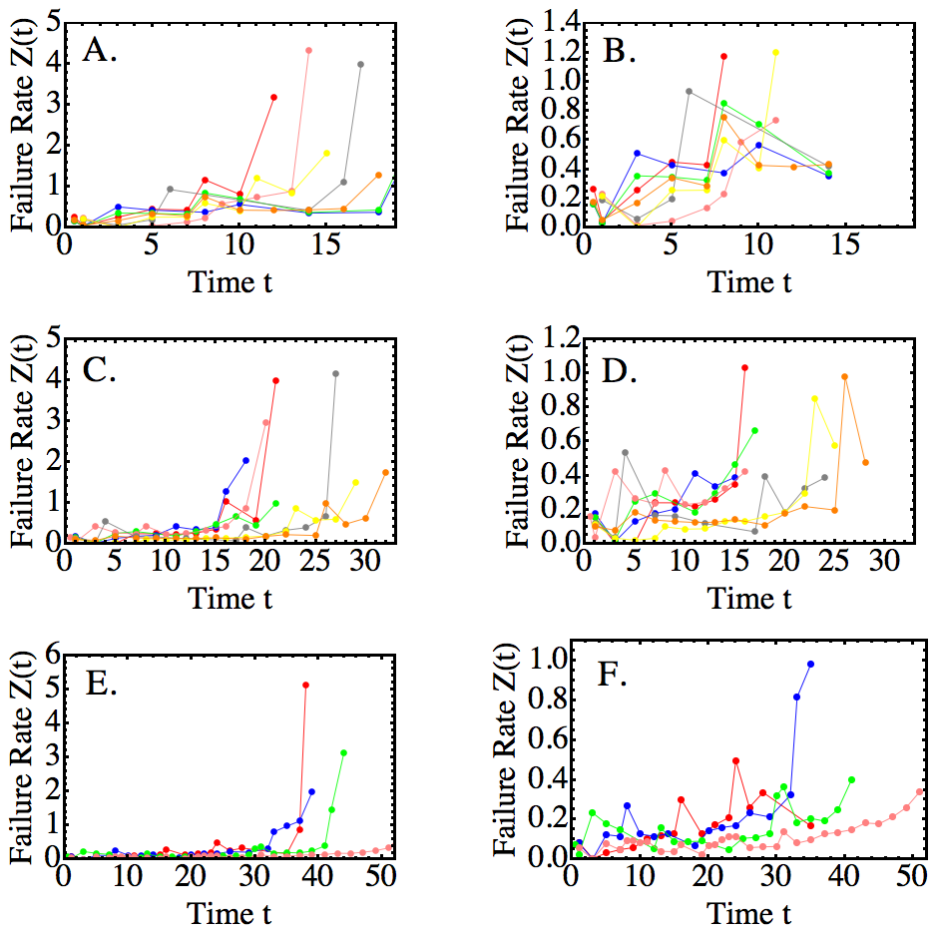
Failure Rate Kinetics of Long-term Repopulating HSCs. To facilitate visualization, failure rate kinetics (vertical axes: each colored line-scatter curve represents the failure rates of the total output of an individual HSC) were displayed in three non-classifying groups (rows A–B, C–D, E–F) and at two levels of resolution (full kinetics in column A, C, E; 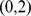-truncated kinetics in column B, D, F) over time (horizontal axes). The full kinetics show that the failure rates increase strongly as the lifespan is approached. We called this behavior the “extinction transition”. The 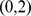-truncated kinetics illustrate the variability, and a tendency to slowly increase, of the failure rates prior to reaching the extinction transition.

The failure rate is defined as the ratio of failure density divided by the probability of reliable operation (which equals the negative rate of change over time of the logarithm of the reliability [39]; also compare M&M, “Reliability Theory”). We applied this definition to the reliability kinetics of the clone, each mature subpopulation and the predicted reliability of the HSC pool to obtain the respective failure rate kinetics (Figure 3, D and Algorithm 1, Line 11). The main observation is that, for all HSC clones, the failure rate kinetics of all populations, and the clone as a whole, increase sharply towards the end of clonal life. The overall behavior is valid for HSCs of all lifespans (Figure 4, A, C, E; also compare Theorems 1 and 2, and Lemma 1). The onset of the increase, which we called extinction transition, coincides with the onset of the increase in the predicted failure rate kinetic of the HSC population. However, the HSC failure rate increases more rapidly than those of the mature cell populations (Figure 3, D). This suggests that clonal extinction is due to an event that affects the HSC population as a whole and, likely, synchronously.

### Repopulation Failure Rates are Mean-Reverting

To better understand the failure behavior of the clonal HSC population before the extinction transition, we looked at truncated failure rate kinetics. We defined a failure rate kinetic as 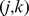-truncated, if 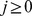 elements are removed from the beginning and 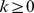 from the end of the time series, respectively. Hence, the full time series is 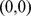-truncated, while 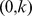-truncation means removing the last 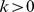 elements of the time series. For all repopulation kinetics in our database (compare Figure 4 for representative examples of different lifespans; also see Figure S1 of Text S1), we analyzed the behavior of the (0,2)-truncated failure rates (Figure 4, B, D, F).

**Figure 5:**
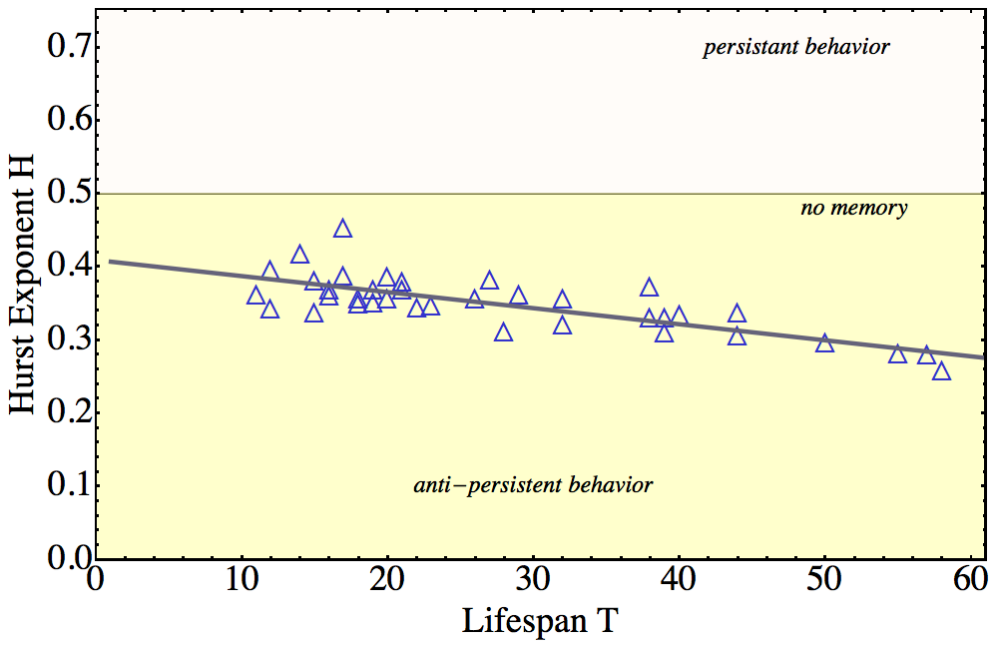
Hurst Exponents of the Failure Rate Kinetics of Long-term Repopulating HSCs. Plotted are the Hurst exponents (plot symbol: blue triangles; values vertical axis) of the failure rate kinetics of HSCs with lifespans 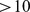 and 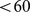 months (horizontal axis). Calculations were performed using Algorithm 0 (compare Table S1 in Text S1). All exponents are 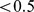, thus falling into the region of anti-persistent behavior (defined by Hurst values 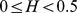 (light-yellow region)) and not into the region of persistent behavior (defined by 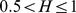 (light-pink region (only displayed up to 0.7 to enhance visibility of the data))). Our previous results [8] that past values of an HSC's repopulation kinetic predict future values, had suggested the hypothesis that Hurst exponents of the failure rates would either be greater, or less, than 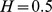. The value 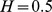 is traditionally interpreted as “no memory” of past behavior in future behavior (horizontal line marked “no memory”). The data shown then suggest that, mechanistically, anti-persistence plays a role in controlling clonal growth. The 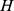 values obtained from our experimental data were fitted to the line 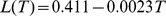 as a function of lifespan 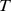 (gray solid line through the data). Goodness-of-Fit was determined using the Akaike Information Criterion (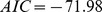). The parameter estimates were highly significant (intercept estimate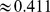, standard error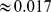, p-value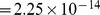; slope estimate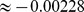, standard error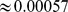, p-value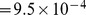). The extension of the fitted line to include lifespans 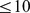 only serves visualization purposes, since we only considered HSCs with lifespans 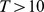 months. The negative slope of the linear fit predicts that anti-persistent behavior in the failure rate kinetics is more pronounced for longer-lived long-term repopulating HSCs than for shorter-lived long-term repopulating HSCs.

We had previously shown that past values in HSC repopulation kinetics predict future values [8]. In other words, memory of past behaviors influences the long-term behavior of repopulation kinetics. We now asked, if memory effects could be shown in the failure rate kinetics. To find out, we calculated the Hurst exponent [49], [52], [53] of each 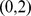-truncated failure rate kinetic (Figure 5; compare section “Computation of Hurst Exponent” in M&M; for values compare Table S1 of Text S1). The Hurst exponent method is a statistical approach for finding long-term memory patterns in a time series. Evidence of memory is defined by the inequality 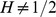, where 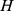 denotes the Hurst exponent. If 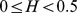, the behavior of a time-series is characterized by a pattern of reversions to a mean, where decreases/increases are followed by increases/decreases. Such a pattern is called “anti-persistent” behavior, which is considered stronger the closer 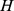 is to 0. When 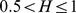, past time series values influence future values either only in the upward, or downward, direction, i.e. increases/decreases follow increases/decreases. This pattern is aptly called “persistent behavior”. Persistence is considered stronger the closer 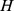 is to 1. Values close to 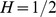 are interpreted as evidence that no relationship exists between past and future values of a time series. Since we had previously shown that past values of HSC repopulation kinetics predict future values [8], we could hypothesize that we might find values of 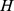 significantly different from 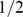 in failure rate kinetics.

To properly conducted Hurst exponent analysis, for example, using a standard approach such as the rescaled range or R/S method, knowledge of the average behavior of all sufficiently large segments of the data is required. Because clonal repopulation kinetics have a deterministic core behavior of ballistic shape [8], we could determine averages based on a deterministic failure rate kinetic for each clone (compare Theorem 1). Plotting the experimental failure rate data together with the deterministic failure rate kinetic showed that the former alternate around the latter (data not shown). Using the additional information available in the framework of HSCs, we could overcome the problem of small time series, similar to approaches in financial market analyses, where standard methods require modifications [54], [55]. Specifically, we used the ballistic trend of repopulation data as a domain-specific mean in the Hurst approach, instead of the uniform mean usually applied. For all clones, the Hurst exponents had median value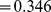 (compare Figure 5; Wilcoxon test, significant difference of the median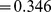 to 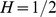, 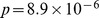). Therefore, the values of 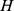 indicated that the failure rate kinetics of HSCs show anti-persistent memory behavior.

Anti-persistence describes a long-range memory behavior of a time series [52], [53], where increases in value are followed by decreases and decreases by increases, as opposed to increases/decreases following increases/decreases, as would be the case for persistent behavior. Using the traditional interpretation, the anti-persistent behavior in the failure rate kinetic of an HSC indicates that the “noise” of the failure rate data follows a long-term pattern that is informative about the biology of HSCs. This pattern suggested the presence of a mean-reverting process in the context of failure kinetics. Therefore, we next considered the possibility that mean-reverting behavior of the failure rate could, biologically, indicate the effects of repair mechanisms acting to decrease the failure rate following increases. In this model, fluctuations in the failure rates, as obtained from measurements of clonal repopulation kinetics, are viewed as indicators for the successive interaction of failure generation and repair, a marked anti-persistent behavior. What is missing, is a quantitative rationale for repair - acting at the cell level, but derived from cell population data.

### Iterative Model of Mean-reverting HSC Failure Rates

Because we found evidence of mean-reversion, we asked how the 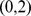-truncated failure rates would fit to realizations of the proto-type mean-reverting process, the Ornstein-Uhlenbeck process [56], [57]. The benefits of linking the HSC failure rates to this process are: (a) A quantitative rationale for repair, in the form of a failure dissipation rate; (b) An iterative model for simulating 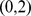-truncated failure rate kinetics based on clonal repopulation data.

We showed numerically that the weighted sum of the variance-adjusted rate of change plus the standardized rate of each 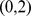-truncated failure rate kinetic could be regressed to the rate of change of noise in the data. Mathematically:
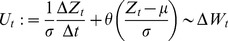
(2)Both sides of the similarity eq 2 can be determined from the data. 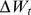, on the right-hand side, represents the discrete rate of change of noise isolated from the data (for normally distributed noise, 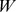 is called a “Wiener process”). 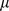 and 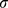 denote the mean and standard deviation of the 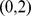-truncated failure rate kinetic, respectively. 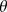 denotes a positive weight parameter, called the “dissipation rate”. Because the dissipation rate occurs in the context of failure kinetics, we interpreted 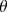 as a quantitative indicator of repair activity - implying that repair could be modeled mechanistically as a dissipation of failure. Eq 2 is equivalent to:
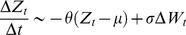
(3)
Eq 3 is a discrete form of the Ornstein-Uhlenbeck stochastic differential equation [56], [57]. Its solution, called Ornstein-Uhlenbeck process, is well-known as the prototypical mean-reverting process.

Our numerical analyses showed that 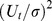 had a non-linear fit of the form 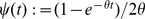. The values of the weight 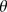, calculated from experimental data, were specific for individual HSCs. Furthermore, we found that in distribution, 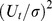 is similar to 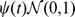, where the second factor denotes the standardized normal distribution with mean 0 and standard deviation 1.

In Theorem 2, we proved that the deterministic repopulation kinetic [8] gives rise to a deterministic differential equation for the failure rate. This equation is formally similar to eq 3, without the noise term. We considered eq 3, therefore, as the approximate Ornstein-Uhlenbeck representation for the 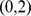-truncated experimental failure rate data.

Together, these findings independently support the prediction, established earlier by Hurst analysis that the truncated failure rate kinetic of an HSC is mean-reverting. We could conclude that the 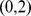-truncated failure rate kinetics can be simulated by an Ornstein-Uhlenbeck model [58], [59] using the iterative scheme:
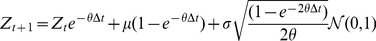
(4)We now asked, if eq 4 could be a model for the full, not only the 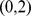-truncated failure rate kinetics. To answer this question in light of the extinction transition seen in the data (sharp increase of failure rates near the end of clonal life), we needed to explain how the mean-reversion property may break down. This required a closer look at the behavior of the parameters 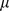, 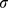 and, in particular, 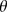, the quantitative indicator of population level repair activity through dissipation of failure.

### Failure Dissipation and Imperfect HSC Repair

**Figure 6:**
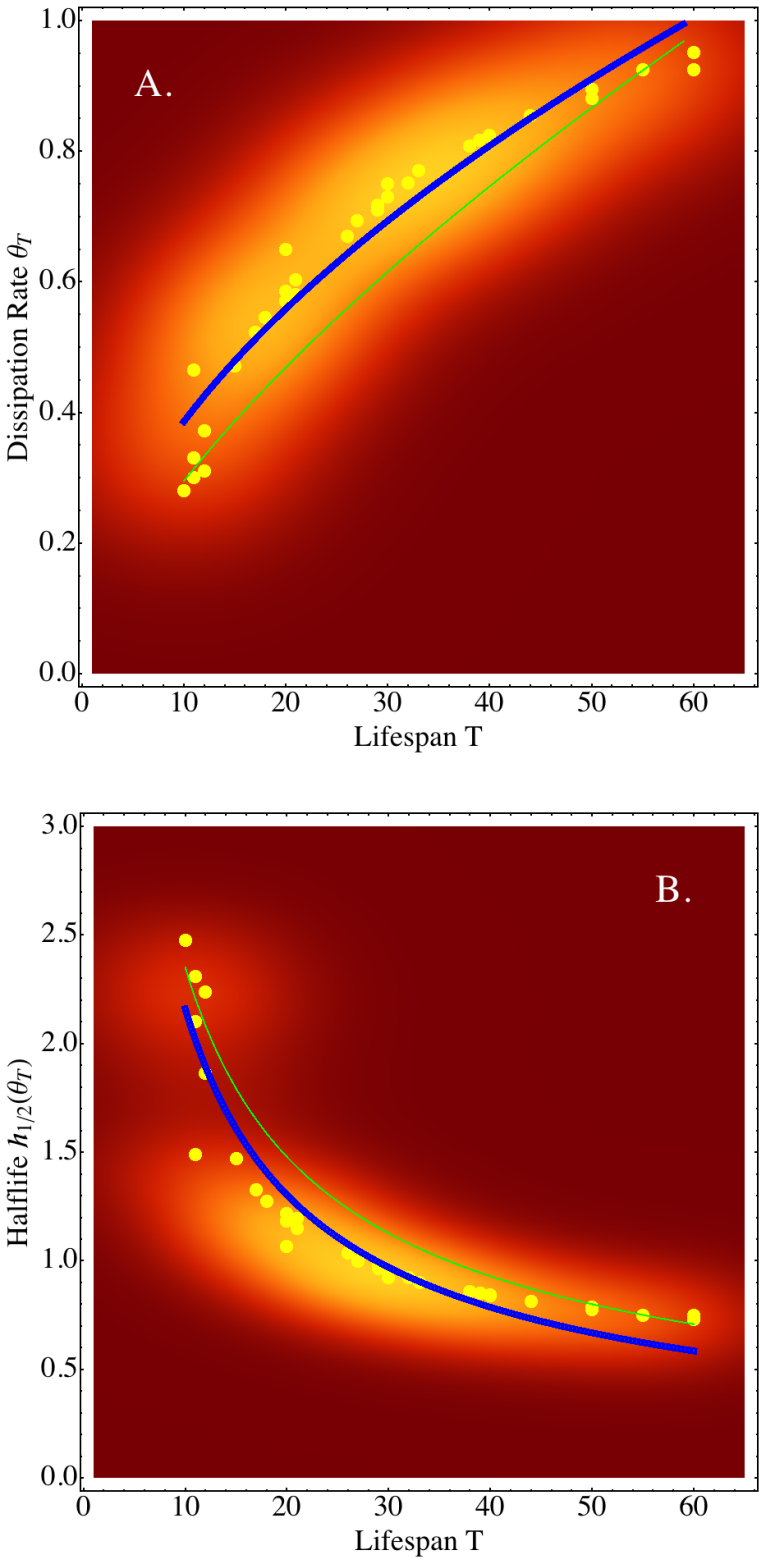
Failures are Dissipated More Slowly in Shorter-lived HSCs than in Longer-lived HSCs. A: We determined the dissipation rates 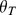 (yellow dots; vertical axis) relative to the lifespan 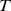 (horizontal axis) using 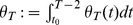 for 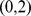-truncated failure rates. The lower bound 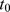 of the integrand was derived in Theorem 2. To highlight the general tendency in the data, not implying any dependencies of consecutive data points, we fitted the data to a non-linear model 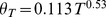 (blue line; goodness-of-fit Akaike Information Criterion: 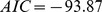); parameter p-values 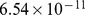 and 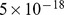). Calculation of 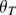 by regressing to normal noise produced a slightly lower exponent 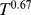 (fitted curve indicated by green line). B: Half-lives of dissipation rates (yellow dots; vertical axis) relative to individual lifespans (horizontal axis). To highlight the general tendency in the data, we fitted the data to a non-linear model 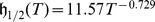 (blue line; goodness-of-fit Akaike Information Criterion: 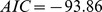; parameter p-values 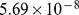 and 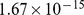, respectively). The model of half-lives obtained from experimental data (green line) is shown for comparison. In both graphics A and B, we used contour plots of the respective data sets 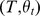 and 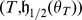, respectively, as background.

We first asked what the properties of 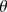 are. The Ornstein-Uhlenbeck formulation (eq 3) allowed us to assess HSC repair efficiency from our clonal repopulation data. We broadly defined “repair” as the total of repair mechanisms available to HSCs. The dissipation rate 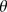 quantitates the strength of the restoring forces as the rate with which the system variable, in our case the failure rate, reverts toward an average behavior 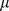. We, therefore, could consider the dissipation of failures as evidence of repair activity.


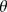
 quantifies how rapidly the clonal system reverts back in the direction of 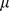-equilibrium. Analysis of the dissipation rate for our experimental data showed that 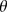 depends on the lifespan 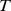. Specifically, determining each 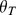 by fitting to a noisy process (eq 2) and plotting the results as points 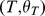, suggested that the dissipation rate has a non-linearly increasing tendency when increasing, but fixed, lifespans are considered (compare Figure 6, A; green curve). Explicitly, we found:
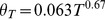
(5)(compare Figure 6, A; green curve; goodness-of-fit: Akaike Information Criterion 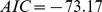; parameter p-values 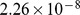 and 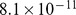, respectively). It is important to understand that eq 5 represents a tendency, not a dependency, among the dissipation rates of repopulation kinetics for independent time-to-failure values 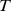. However, going back to a remark at the beginning, the time-to-failure is a function of load which, as noted, comprises two components relating to peripheral demand, and demands due to disease or injury, respectively. Biologically, load may affect, in parallel, all accessible HSCs in the HSC compartment of a single host. Therefore, if 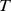 is parametrized by load, eq 5, and the eq 6 below, may be interpreted as a power law, dependent on load exposure. This suggested that 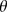 may be dependent on “running” time, as well.

To find out, we used an analytical approach that took advantage of the deterministic behavior of HSC repopulation kinetics. In Theorem 2, we showed that an analytical definition of 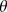 can be found as a function of 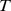 and 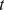, denoted 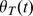, based on the ballistic model of repopulation kinetics developed by us previously. To see if behavior similar to eq 5 can be found analytically, we averaged 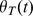, defined by 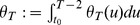, where integration extends over bounds that are analogous to 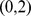-truncation of failure rates (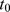 is explicitly given in Theorem 2). We found that the dissipation rates derived analytically or from data have similar properties (compare Figure 6, A). As before (compare eq 5), we fitted the resulting set of 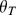 values to a non-linear model:
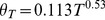
(6)(compare Figure 6, A; blue curve; goodness-of-fit: Akaike Information Criterion 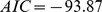; parameter p-values 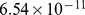 and 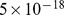, respectively). Both approaches show the same tendency of the dissipation rate to increase as a point-wise function of 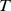. Hierarchical cluster analysis showed that the (lifespan, dissipation rate) data 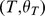 separate into three clusters. The dissipation rates of the three cluster centroids 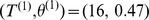, 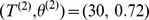 and 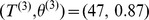 are significantly different (Wilcoxon Test, Bonferroni corrected p-values: 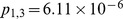, 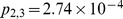, 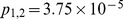).

Together, our findings suggested that HSCs of different lifespans may differ in their ability to utilize repair mechanisms. We used the formulae developed in the proof of Theorem 1 to compare the dynamics of failure generation and failure dissipation (repair). As stated above, we found that, for the deterministic repopulation kinetics, the dissipation rate is a function of time 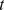 and the lifespan 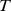, i.e. 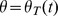, for 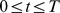. 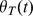 equals the negative logarithmic derivative of the failure density function. As shown in the proof of Theorem 2, 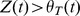 for all 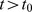, with 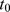 that can be calculated from data. This means that, throughout most of the lifespan period, failures are generated at a higher rate than they are dissipated. Therefore, we could use the inequality 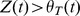 to quantitatively define “imperfect repair” and to ascertain that repair activity is largely insufficient to compensate for the rates at which failures are generated. The analytical treatment supported the notion that failures should accumulate in the long-run.

The mathematical analysis also predicted that, during the initial expansion period of a clone, quantitated by 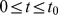, repair capacity exceeds the rate at which failures are generated. Quantitatively, 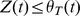 for 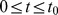. In particular, our theory predicts that each HSC starts “its” clone with a non-zero “initial damage load” 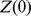 [40]–[42]. The “initial damage load” can be calculated for each repopulation kinetic. Its precise biological meaning and, particularly, its developmental origins, will need to be determined experimentally. The switch from higher repair capacity to 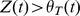 may be attributable to the diluting effects of clone size on the programmed, epigenetically heritable, repair capacity of the founder HSC.

### Why Some HSCs Live Longer Than Others

Evidence favoring the hypothesis that shorter lived HSCs may be less efficient in dissipating the effects of failures than longer lived HSCs, can be biologically interpreted by placing the dissipation rate in a time-context. The time-context is given by the half-life of the dissipation rate. The half-life is defined by:
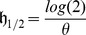
(7)


The half-life data (compare Figure 6, B) show a tendency, where failure rate increases dissipate more rapidly in long-term repopulating HSCs with higher lifespans 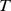. This holds independently of the manner in which 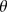 values were obtained, either analytically or from data. Mathematically:
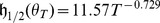
(8)(goodness-of-fit: Akaike Information Criterion 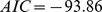; parameter p-values 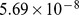 and 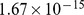, respectively). Hierarchical cluster analysis showed that the (lifespan, half-life) data 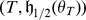 separate into three clusters. The half-lives of the three cluster centroids 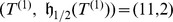, 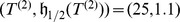, 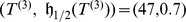 are significantly different (Wilcoxon Test; Bonferroni corrected p-values: 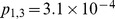, 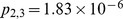, 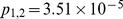). Like eq's 5 and 6, the relationship eq 8 must be interpreted with care as 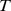 is a time-to-failure variable and should not be confused with a continuum.

The data show larger failure dissipation rate half-lives for HSCs with shorter lifespans, while smaller half-lives associate with longer life. A possible interpretation is that repairs may occur less frequently in shorter lived HSCs than in longer lived HSCs. According to Theorem 1 shorter lived HSCs may have to counteract higher initial damage loads (as suggested by the higher initial values of 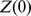 for smaller lifespan values). Together, these findings may explain the larger and longer-lasting “peaks” and “valleys” seen in the failure rate kinetics of shorter lived HSCs (Figure 4).

### Break-down of Mean-Reversion and Clonal Extinction

**Figure 7:**
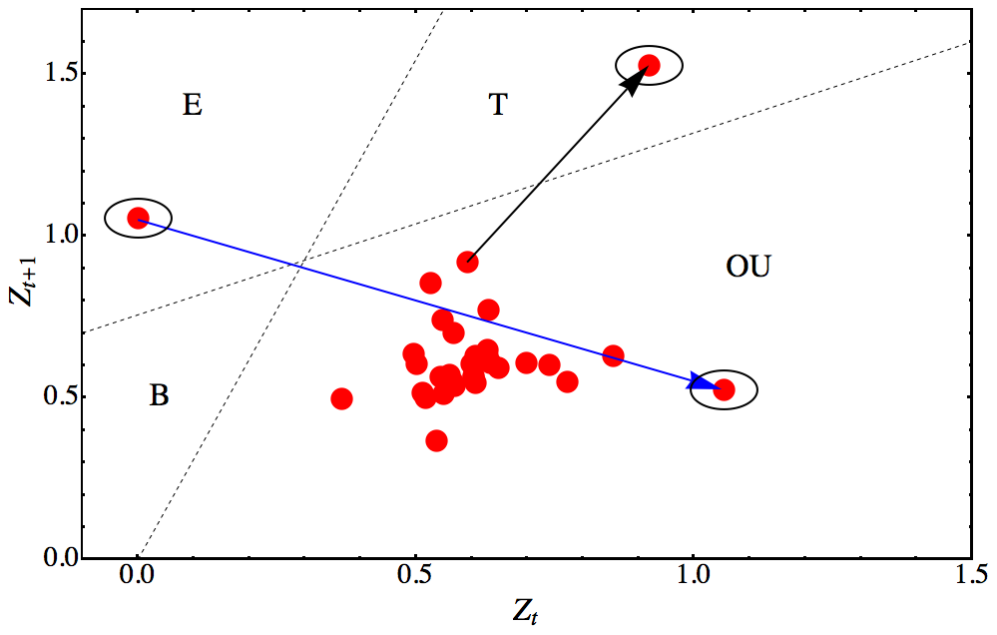
Failure Rate Phase Space Regimes of Long-term Repopulating HSCs. Phase space plot of the failure rate kinetic of a long-term repopulating HSC with long lifespan of 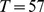 months. Points 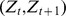 in the plot (red) represent successive failure rates calculated every 2 months. To facilitate visualization, regions were separated by dotted lines. After initial expansion (region E, circled point), the kinetic transitions (blue arrow) into a regime (region OU), where it remains for most of clonal life. The behavior in region 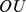 is governed by an Ornstein-Uhlenbeck iterative process (compare eq 4). The end of clonal life is indicated by the transition (black arrow) from region 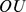 to the “terminal” absorbing point in region 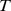 (circled point). Region B is not visited by the dynamic trajectory and, therefore, empty.

An important observation common to all failure rate kinetics is that, near the end of clonal life, the failure rates strongly increase. When we compared actual failure rate kinetics with kinetics generated by simulation using the experimentally derived parameters in eq 4, we noticed that the terminal behavior of the experimental failure rate departed significantly from the mean-reverting characteristic of the simulated rates (for more detailed discussion see below). This suggested that regime-breaking may characterize the extinction transition. “Regimes” is standard terminology to describe disjoint regions in the phase space of a dynamical system such that transitions between the regions are rare (compare Figure 7 for a representative example). We, thus, analyzed the regimes of the phase space of experimental HSC failure rate kinetics.

The phase space trajectory of an HSC's failure rate kinetic starts in the initial engraftment regime (Figure 7, region E). Here, it remains for up to 3 months of the HSC's life. Then, the trajectory escapes to the region where failure rates are governed by mean-reversion (Figure 7, region OU). It remains in this regime for most of the clonal life - first contracting, then slowly expanding. At some point, the phase space trajectory transitions to a third regime (Figure 7, region T), where the clone becomes extinct. Therefore, regime-breaking is associated with clonal extinction.

**Figure 8:**
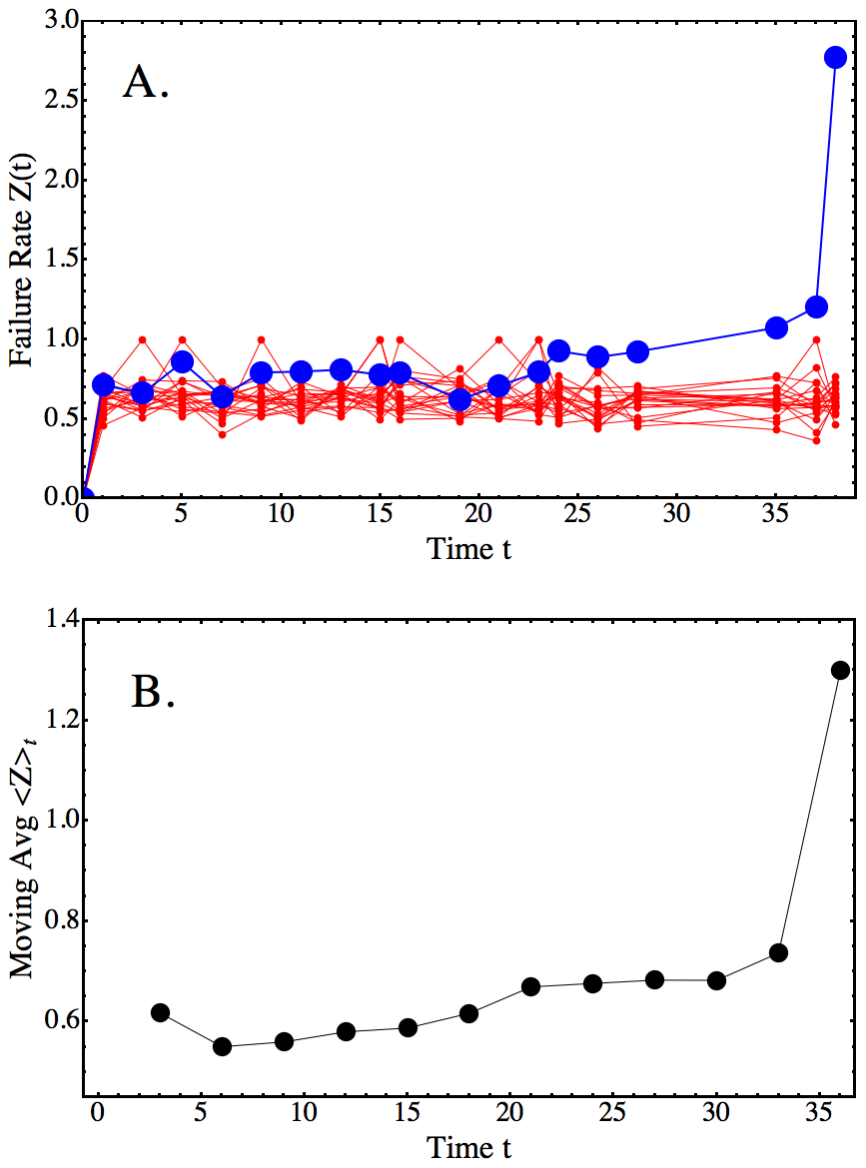
Breakdown of Mean-Reverting Behavior in the Failure Rate Kinetics of Long-term Repopulating HSCs. A: An experimental failure rate kinetic (blue scatter-line plot; values 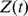 vertical axis) compared to the kinetics of 100 realizations (thin red lines) of an Ornstein-Uhlenbeck process over the lifespan period (horizontal axis) of a clone with lifespan 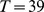 months. The realizations of the process were obtained using the iteration schema in eq 4. The same values of 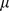, 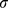 and 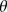 as in the experimental data were used. For simplicity, the initial condition was set at 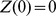 for 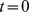 (equivalent to assuming a load-free transplant). The important observation is that without additional conditions on the Ornstein-Uhlenbeck process, the expected behavior of the kinetic generated from data (blue curve) will not occur. B: The moving average (vertical axis; window size = 6) of the same failure rate kinetic as in Part A (blue line-scatter curve) reveals that the parameter 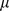 increases slowly during the mean-reverting regime (raw moving average data (denoted “Moving Avg 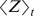
”) are in black). The slow increase changes to rapidly increasing failure rates at around 82% of the lifespan. Both behaviors combine into the model of equation 9 with parameters 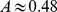, 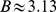 and 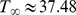 (p-values = 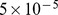, 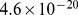, 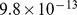, respectively; 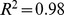).

We next established the conditions under which the mean-reverting regime breaks. As discussed above, comparison of the experimental failure rate kinetics with realizations of the iterated Ornstein-Uhlenbeck process (eq 4) showed that the simulated process diverges from the experimental data in the terminal regime (Figure 8, Part A). The simulated failure rates continue as before, but the experimental failure rates rise sharply. This behavior could be changed, and brought closer to the experimental data, when a further constraint was added.

Specifically, we asked whether the mean of the experimental failure rates is a constant or changes in time. Analysis of the 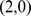-truncated failure rate kinetics of each clone using moving averages showed that the parameter 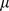, the mean failure rate, increases in a well-defined pattern that occurs in all kinetics (compare an example in Figure 8, B). Though this pattern of increasing mean is present in the raw failure rate data, the change there is subtle, but cumulative. It is enhanced and, thus, becomes more recognizable if moving averages are used (for short lifespans only small windows are needed; larger windows (up to 5 months) are required for longer lifespans). Using non-linear fitting, we found that the succession of moving averages, labelled 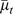, increases over time as:
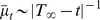
(9)The critical limit 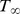 is slightly smaller than the time-to-failure, or lifespan, 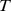 of the clone. This result obtained from data analysis has an analytical counterpart. In Theorem 1, we showed that the ballistic model of HSC repopulation kinetics [8] leads to a deterministic formula for the failure rate function of any HSC clone, such that, according to Lemma 1, 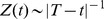 in the limit 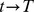. Therefore, the analytically derived result matches the behavior extracted from data in eq 9.

We could, thus, conclude that as more self-renewals occur, repair mechanisms must revert to larger failure rates means in daughter HSCs. Biologically, the increasing mean suggests that damage accumulates over successive generations, likely due to imperfect repair.

## Discussion

Clonal hematopoiesis begins with a single HSC and ends with its loss after months to years [4]–[8]. During this period of time - the lifespan -, the genetic/epigenetic program of the original HSC is replicated to many daughter HSCs. In HSC self-renewal, discrepancies between replicates should be vanishingly small. Yet, clones extinguish after a limited time, suggesting that self-renewal may not be perfectly reliable in the long-run.

Working from repopulation kinetics, we needed to develop quantitative measures of reliability, failure and repair that capture events of magnitudes that approach population size. Our data show that increases in failure rates associate with increasing population-wide failure loads, and decreases relate to dissipative effects of the collective repair strength. Together, the fluctuation patterns of the failure rate kinetics characterize the summary dynamics of microscopic failure and repair events at the macroscopic level of clonal HSC populations.

Our approach does not depend on particular failure sources and repair mechanisms. An advantage, therefore, is that our current understanding of stem cell Omics is not limiting. Rather, reliability theory could open new avenues for interpreting longitudinal network data. For example, we make the prediction that repair capability stays approximately constant through the lifespan of an individual HSC. Constancy does not mean that individual repair mechanisms are unaffected by failure inducing processes. Instead, the function of a deteriorating repair mechanism could be taken over by an alternative pathway.

We predict that the repopulation failure rate kinetics stay at low levels for a long time, but will never revert to zero failure rate. This supports the conclusion that failures are continuously generated, but are never completely cleared. Indeed, failure rates increase slowly, indicating that failures accumulate. Evidence of failure accumulation is not only seen in our experimental data. It also followed by mathematical proof (M&M section; Theorem 2) for the deterministic failure rate kinetics derived from our previously developed ballistic model [8].

Daughter HSCs of successive generations may, thus, carry an increasing failure load - consistent with previous experimental findings on the aging of HSCs [60]. As shown here, increasing failure load coupled with constant repair capacity is key to explaining the differences in lifespans of HSCs in a single host in the absence of strong extrinsic perturbations (e.g., expression of the Wnt inhibitor Dickkopf 1 [38]). These predictions could be tested experimentally using longitudinal genome studies comparing genetic networks in myeloid-biased (My-bi) and lymphoid-biased (Ly-bi) HSCs [47]. The former are typically longer lived than the latter. Reliability analysis may aid in predicting universal check-points (such as 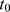 in Theorem 2) to meaningfully time longitudinal genome studies of My-bi and Ly-bi HSCs. So far, the genome of My-bi HSCs has only been mined at isolated instances [47], [61]. Timing of genomic analyses is experimentally challenging, since HSCs are rare relative to mature blood cell populations.

We showed that the failure rate is stable, but “noisy”. This “noise”, properly classified using rescaled range, or Hurst, analysis [53] shows how stability is generated. The experimental failure rate kinetics alternate irregularly around the deterministic failure rate component, which we used as the mean value in our rescaled range analysis of the data. We found that the Hurst exponent is 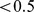 for the failure rate kinetics of all long-term repopulating HSCs. HSCs with lifespans greater than one mouse life have smaller exponents than for lifespans below one mouse life. Using non-linear time series theory, we could conclude that an anti-persistent, or mean-reverting, regime governs the time period where failure rates are at a minimum. The implication is that the interaction between failure-generating mechanisms, such as HSC self-renewal divisions, and failure-dissipating processes, or repairs, creates a self-organized failure-repair equilibrium. An HSC clone exits the equilibrium state, and becomes extinct shortly there-after, when failure load has accumulated sufficiently to surpass repair capacity.

Our reliability analysis of HSCs has implications on aging in HSCs [2], [45], [62] and in other non-homogenous, hierarchically organized, cell systems. The elegant general theory of aging developed by the Gavrilovs [40]–[42] - tested extensively for populations of organisms but less for those of cells [43], [44] - poses that aging systems have three properties: redundancy, initial damage load, and redundancy depletion. For HSCs, redundancy emerges over time as a function of self-renewals. The declining quality of genome replicates, due to imperfect repair as predicted here, quantifies the rate of redundancy depletion in HSCs. The mathematical results of this paper predict that the failure dissipation rate determined from our data provides a quantitative measure of “progressive damage load” starting from an HSC-specific “initial damage load”. However, what constitutes “initial damage load” in the biology of HSCs, and what its sources are, must first be investigated experimentally - primarily to address the question of how HSCs are programmed during early development.

## Materials and Methods

### Clonal Analysis

Freshly explanted BM cells were transplanted in limiting dilution into lethally irradiated CD45 congenic hosts exactly as described [45]–[47], [63]. Each host received on average 0.2–0.5 HSCs together with 2×transplanted BM as a source of radio-protecting cells. Mice were bled in regular intervals and the % myeloid and lymphoid cells amongst the donor-type cells were measured by Flow cytometry. All experiments were approved by the IACUC.

### Software

We used Mathematica version 8.0.1 (Wolfram Research, Inc) for numerical mathematics and computer simulations. R version 2.12.2 and Instat version 3 (GraphPad, Inc) were used for all statistical analyses. Figures were generated using Mathematica version 8.0.1 and edited using GIMP version 2.8.3. The manuscript was written in LaTeX2e using GNU Emacs version 23.3.50 (Free Software Foundation).

### Reliability Theory

The subject of reliability theory is to determine the length of time for which a system is capable of bearing “load” given its material “strength”. Mathematically, system reliability versus unreliability at time 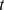 is quantitated by the respective inequalities between strength 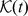 and load 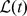:
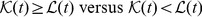
(10)Strength and load are non-negative functions of time, i.e. 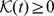 and 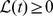. A system's time-to-failure 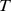 is defined as the earliest point in time for which load exceeds strength, i.e.:
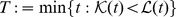
(11)For repairable systems, multiple occurrences of 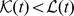 are possible, only to be reset by the repair process to some level of reliable operations, i.e. 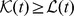. Hence, the operation of a repairable system will generate a sequence of time-to-failure values. By contrast, in an unrepairable system, the first time-to-failure is also the last.

Though load and strength can be deterministic, it is advantageous to consider the general case where the strength-load inequality (compare eq 10) is subject to uncertainty. Hence, system reliability is usually defined by a probability measure 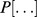 for events 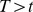 (colloquially: “time-to-failure not yet reached”):
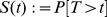
(12)The explicit reference to the strength-load inequality 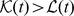 can be suppressed due to the definition of the time-to-failure (eq 11). The definition of probabilities implies that 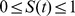.

The failure probability is defined as the conjugate probability:
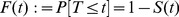
(13)The rate of change of the failure probability over time is used to define the failure density:
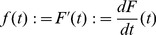
(14)This definition requires that the reliability 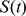 is a differentiable function of time 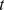.

The rate of system failure, or failure rate, is determined by the ratio of the failure density to the system's reliability, provided that 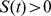:
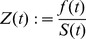
(15)Due to the properties of the reliability and the failure density, it follows that 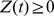 for all permissible 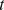. Using eqs 14 and 13 in eq 15 shows that the failure rate can also be represented by the derivative of the negative logarithm of the reliability.

### Computation of Reliability and Failure Measures

In practical applications, a system's reliability is determined based on field measurements of a particular system variable associated with well-defined, system-specific, time-to-failure conditions. From these measurements, one attempts to form a discrete empirical distribution as a time series 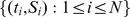, where 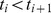, normalized such that 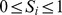, and 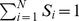 (compare Algorithm 1).

Statistical distribution fitting with appropriate goodness-of-fit measures may identify a suitable closed-form model 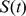 (such as in eq 12), so that 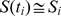. In this case, a system's reliability and failure rate evolution can be described using probability, dynamical systems theory, and stochastic processes. We show in the main narrative that the failure rate of the repopulation kinetics of hematopoietic stem cells follow an Ornstein-Uhlenbeck stochastic process.

Time series representing the evolution of discrete failure probabilities and failure densities can be obtained by point-wise application of eqs 13 and 14 (as used in Algorithm 1). In the case of the failure density, the differences operator is used to approximate differentiation. This operator is defined by
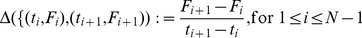
(16)


We computed discrete failure rates as the ratio of density to reliability, as introduced in eq 15. This approach is computationally more efficient and more transparent than using the numerical derivative of the negative logarithm of the reliability sequences.

### Computation of Hurst Exponent

To make predictions about the long-term reliability of water reservoirs, Harold Hurst introduced a new statistic, called the rescaled range. This statistic is determined by forming the ratio of the difference between the water level extremes over a long time period, called the range, to the standard deviation from the mean water inflow over the same time period, but using sub-divisions of the time scale into smaller segments [64].

Because of its generality, the rescaled range, or R/S, statistic can be used in many different contexts - with appropriate reinterpretation of time scales and measured entities. Hurst's original finding, expanded by Mandelbrot and collaborators [49], [52], [53], was that, in the limit, the average rescaled range over time periods of increasing size 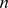, behaves like a fixed power of 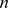:
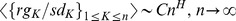
(17)

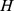
 is called the Hurst exponent. An algorithm for determining the rescaled range statistic and the empirical Hurst estimate is given below (Algorithm 2). Explanations for the notation used above are given there. Implementations of the R/S and other methods for estimating the Hurst exponent are available in the statistical programming language R.

**Algorithm 2. **Algorithm for calculating the rescaled range statistic and estimating the Hurst exponent 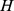 **.** The notations used are the same as in Algorithm 1. In addition the functions Max() and Min() refer to the maximum or minimum of a data set 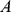 respectively. The notation 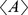 denotes the uniform sample mean of 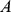. The function LinModFit(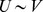) linearly regresses the predictor data 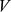 on the response data 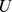 with 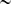 being used as in the statistical programming language R to express a potential relationship between data. 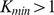 is a minimum lower bound on time series size to conduct meaningful analyses.

**input**: A time series 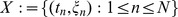 of length 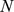, with 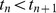, 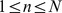.

**output**: A sequence RS of rescaled range values of 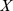 and a number 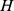 representing an estimate of the Hurst exponent.

**1 for** 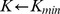 **to** 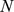 **do**

**2** 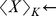 Sum 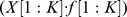; **//** 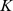 **-th mean with respect to PDF** 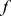;

**3** 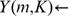 Sum 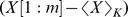; **// cumulative deviation from mean;**

**4** 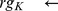 Max 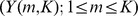 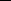 Min 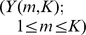; **// range**

**5** 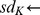 Sum 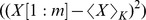; **// standard deviation**;

**6** 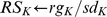; **//** 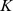 **-th rescaled range**;

**7** 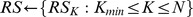; **//rescaled range sequence;**

**8** 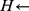 LinModFit (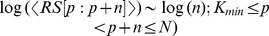;

In applications, the benefit of determining the rescaled range sequence is that we can analyze and interpret data that have no characteristic scale. This is sometimes interpreted as lacking bias introduced by specific measurement scales. The Hurst exponent, 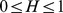, measures the smoothness of (self-similar) time series based on the asymptotics of the rescaled range sequence. 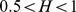 indicates a persistent, or trend-reinforcing, time series. In this case, increases (decreases) of time series values are followed by increases (decreases). This trending increases as 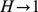. 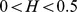 holds for mean-reverting, or anti-persistent, time series. In this case, deviations from the mean lead to reversal of the time series values towards a long-term mean. The “strength” of the mean reversion increases as 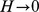. Geometrically, anti-persistent time series will appear more jagged as 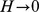. The value 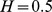 is considered indicative for lack of correlation in the time series: Any values do not inform about future values. In the case of the hematopoietic system, we showed previously that past values of the clonal repopulation kinetics are predictive for future values [8].

### Exact Reliability & Failure Kinetics: Theorem 1

We wished to identify the failure rate kinetics under noise-less conditions. To do this, we could use the deterministic model of clonal repopulation kinetics, denoted 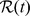 below, that we had developed previously [8] based on experimentally obtained clonal repopulation data.

#### Theorem 1

*Consider the ballistic kinetic* 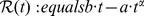 *, with parameters* 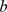 *,* 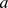 *, and* 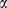 *such that* 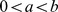 *,* 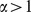 *. Let* 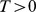 *be such that* 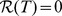 *. Define the rate* 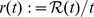 *. The failure rate* 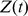 *of the reliability* 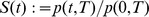 *, where* 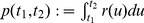 *,* 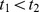 *, equals:*

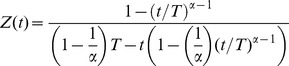
(18)
*and satisfies:*

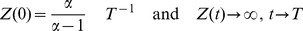
(19)

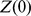
 *is defined as “initial damage load” and depends only on the system parameters.*

*Proof.* From the definition of 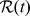 follows 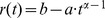. Then:
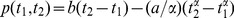
(20)from which follows that 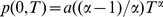. Therefore, using 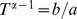, the function 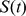 has the form:
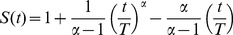
(21)It follows from eq 21 that 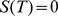 and 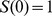. Furthermore, integration shows that we can express 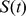 in a normalized form, since:
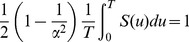
(22)Since 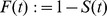 (eq 13), we get:
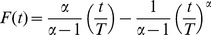
(23)It follows that 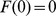 and 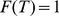. Differentiation with respect to 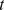 and using eq 14 shows:
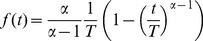
(24)One notes that 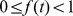 for 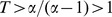. Using eq 15, we find:
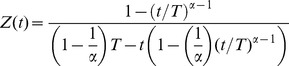
(25)It follows that 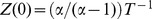. If we write eq 25 in the form 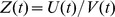, we see that 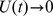 and 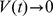 in the limit 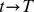. The numerator and denominator are differentiable, so that with the substitution 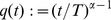:
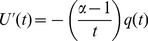
(26)

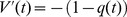
(27)from which follows:
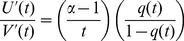
(28)It follows from de l'Hopital's rule that 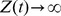, as 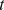 approaches 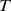 from below.

### Asymptotic Behavior near the Lifespan: Lemma 1

In Theorem 1, we showed that 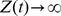 in the time limit approaching the lifespan. The following result details the asymptotic growth properties of the deterministic failure rate kinetic.

#### Lemma 1

*The asymptotic behavior of the repopulation failure rate near the lifespan* 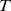 *is:*

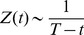
(29)
*where, symbolically, the relationship* 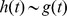 *between two functions* 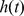 *and* 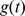 *indicates similarity in the limit.*

*Proof.* Series expansion of 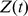 near 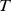 shows:
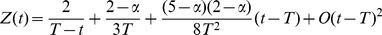
(30)


### Gross Value Distribution of Failure Rates: Lemma 2

We wanted to know the order of magnitude of the “initial damage load” 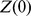 in relation to the lifespan 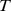. In particular, is there a lower bound on 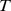
?

#### Lemma 2

*For* 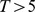 *we have* 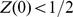 *. Furthermore:* 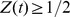 *for* 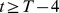 *.*

*Proof.* We have for 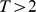:
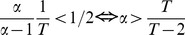
(31)The claim follows from the condition 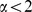. Furthermore, using the asymptotic expansion in the proof of Lemma 1, we get:
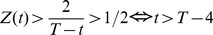
(32)from which the second claim follows.

### Failure Growth and Dissipation: Theorem 2

We wanted to know under which conditions on time 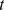 the deterministic failure rate kinetic 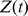 increases and decreases. To find answers, we determined explicitly the rate of change of the noise-less failure rate kinetic derived in Theorem 1. Throughout, we used the simplified notation 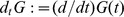 for the derivative of a differentiable function 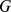 of variable 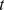.

#### Theorem 2

*The rate of change* 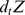 *of the failure rate* 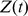 *has the form:*

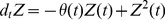
(33)
*with a time-dependent dissipative “frictional force” term* 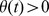 *. The latter is given explicitly by the logarithmic rate of change of the failure density:* 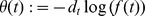 *. Furthermore,* 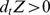 *for* 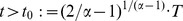 *, implying that* 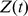 *is strictly increasing for* 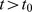 *. Furthermore,* 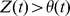 *for* 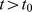 *.*

#### Remark


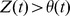
 *for* 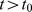 can be interpreted as “imperfect” effects of the dissipative force. This motivated our quantitative definition of “imperfect repair”.

*Proof.* We write 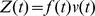 in the form of an intensity with 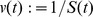. Differentiating, we get:
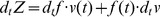
(34)Since 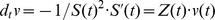, it follows that:
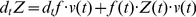
(35)

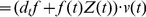
(36)This expression can be rewritten once more to yield:
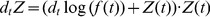
(37)where, explicitly:
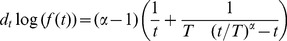
(38)Since 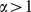 and the second factor is always negative, it follows that 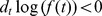 for 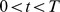. Furthermore, 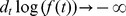 for 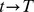 and 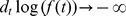 for 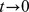.

We now define 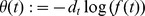 to obtain the general expression for the slope of the failure rate:
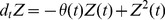
(39)with the time-dependent positive “dissipation”, or retarding “frictional force”, term 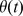. The latter is given explicitly by the logarithmic rate of change of the failure density as shown above.

Since 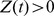, 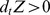 when 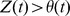 or 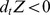 when 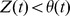. Using the definitions of 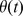 and 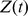, 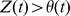 is equivalent to 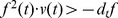 or 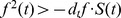. Direct calculation shows that
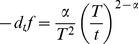
(40)Using eqs 21 and 24 from the proof of Theorem 1, 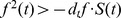 becomes:
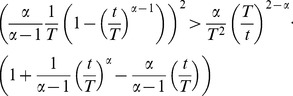
(41)or, equivalently:
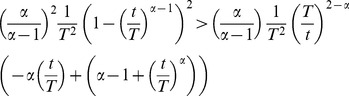
(42)which is equivalent to:
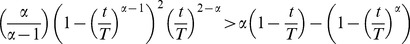
(43)With the substitution 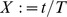, the latter becomes an inequality between two functions denoted 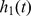 and 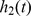, respectively:
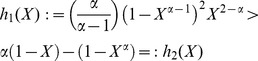
(44)The function 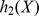 on the right-hand side of inequality 44 is positive for 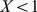, has the value 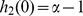 at 0, and decreases strictly monotonically to 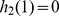. The function 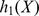 on the left-hand side of inequality 44 also satisfies 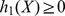 with 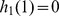, but 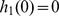. Some manipulation shows that 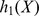 is monotone increasing for 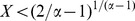 and decreasing for 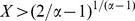. Taking the second derivative of 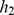 and resubstitution shows that 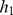 takes a maximum at: 
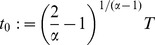
(45)and the inequality 44 is satisfied starting in a neighborhood of 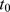, i.e. 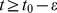. It follows that 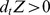, at least from 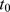 on.

With some algebraic substitutions, eq 33 can be formally rewritten as a deterministic form of the Langevin equation. Specifically:

#### Corollary

*With* 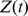 *,* 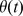 *having the same meaning as in Theorem 2, we can write*

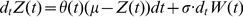
(46)
*with 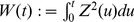 interpreted as a mean-squared variation of* 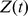.

*Proof.* Replace 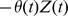 by 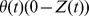 for the exact 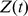. Write 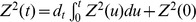. Define the function 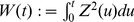 to replace 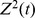 by 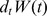.

### Statistical Analyses

To analyze the (lifespan, dissipation rate) and (lifespan, half-life) data, denoted 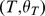 and 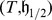, respectively, in the main narrative, we first standardized the data by subtracting the sample mean and dividing by the sample standard deviation column-wise. We used hierarchical cluster analysis on the standardized data to determine the number of clusters and their centroids. Confirmatory analysis was conducted using a standard partitioning approach.

## Supporting Information

Text S1 It is first shown that the empirical estimator of the time-to-failure, or lifespan, of the repopulation capacity of clonal long-term repopulating hematopoietic stem cells is statistically unbiased, and almost efficient relative to a Gumbel distribution of maximum extremes. Together, these properties indicate that the Gumbel distribution makes best use of the information obtainable from serial repopulation experiments. Table S1 contains experimentally determined HSC lifespans, growth and decline rates, and the Hurst exponents calculated using Algorithm 2 (compare Materials & Methods). Figure S1 shows the shapes of clonal repopulation time series obtained experimentally by measuring the percent donor-type cells in blood after transplanting a single HSC.
(PDF)
