# Supplementary material for: Lifespan Differences in Hematopoietic Stem Cells are Due to Imperfect Repair and Unstable Mean-Reversion
Source: PLoS Comput Biol. 2013 Apr 18;9(4):e1003006. doi: 10.1371/journal.pcbi.1003006 (PMC3630147; doi:10.1371/journal.pcbi.1003006)
Supplement: Text S1 — It is first shown that the empirical estimator of the time-to-failure, or lifespan, of the repopulation capacity of clonal long-term repopulating hematopoietic stem cells is statistically unbiased, and almost efficient relative to a Gumbel distribution of maximum extremes. Together, these properties indicate that the Gumbel distribution makes best use of the information obtainable from serial repopulation experiments. Table S1 contains experimentally determined HSC lifespans, growth and decline rates, and the Hurst exponents calculated using Algorithm 2 (compare Materials & Methods). Figure S1 shows the shapes of clonal repopulation time series obtained experimentally by measuring the percent donor-type cells in blood after transplanting a single HSC. (PDF) [file pcbi.1003006.s001.pdf]

Text S1

Unbiased and Almost-efficient Time-to-Failure

We had previously shown [1], using machine-learning techniques, that the HSC lifespan can be predicted with high accuracy using only the first 4 values of the repopulation kinetic. We showed that the predictor of the lifespan T can be expressed through HSC-intrinsic parameters in the form:

$$\hat{T} = (b/a)^{1/(\alpha-1)} \tag{1}$$

The parameters b, a, and α, with $b > a$ and $\alpha > 1$, represent the HSC-specific growth and decline rates, and the degree of slowing down, respectively.

The estimator $\hat{T}$ is unbiased. In the standard notion $E[X]$ for the expectation, or average, of a random variable X, we found that $E[\hat{T} - T] \approx 0.0552$. The value 0.0552 is not significantly different from 0 (p-value: 0.84), which implies the claim.

We asked if the estimator is also efficient. Efficiency means that all, or most, information available from the repopulation kinetics of HSC clones is actually used in the prediction of the time-to-failure of an HSC. We used the Cramer-Rao inequality [2] to determine if the variance of the unbiased estimator $\hat{T}$ is bounded from below by the Fischer information $I[T]$ of the lifespan, or $\mathrm{Var}[\hat{T}] \geq 1/I[T]$. If $\mathrm{Var}[\hat{T}] = 1/I[T]$, the estimator $\hat{T}$ would be "efficient", indicating that information is optimally used.

We calculated the Fischer information $I[T]$ using the estimated probability density function $G_T(u)$ of lifespans:

$$I[T] := \int_0^\infty (d_u G_T)^2 G_T(u) du \tag{2}$$

with $d_x := d/dx$. The sample density is of Gumbel type of maximum extremes [3] with location and scale parameter values 20.5 and 7.77, respectively. The empirical distribution of lifespans and the Gumbel model are not significantly different (Pearson χ^2 test: p-value = 0.93). Using the return period [4], shows that no lifespans greater than 90 months can be expected under this distribution. Approximately 5% of HSCs should have lifespans of 60 months, or higher, and the likelihood of finding HSC lifespans ≥ 70 months is about 2%. This explains why we can routinely find "Methuselah" HSCs in our experiments, but no normal HSCs with a lifespan beyond the estimated upper limit.

We found that $1/I[T] \approx 86$. On the other hand, we found that $\mathrm{Var}[\hat{T}] \approx 99$. Therefore, $\mathrm{Var}[\hat{T}] \cdot I[T] \approx 1.15$, which is slightly larger than the best possible value of 1. Because of this small difference between the actual and target values we considered the estimator $\hat{T}$ "almost efficient". Other potential densities from the same family of distributions as the Gumbel density of maximum extremes yielded values of $\mathrm{Var}[\hat{T}] \cdot I[T]$ larger than 1.15. Accordingly, we concluded that the Gumbel maximum extremes distribution makes best use of the information available from repopulation kinetics.

Table S 1. Parameters of Repopulation Kinetics and Hurst Exponents.

Clone #	T	b	a	b/a	H	Clone #	T	b	a	b/a	H
1	11	44.66	14.08	3.01	0.361	20	22	18.39	4.42	4.15	0.343
2	12	43.35	14.17	3.06	0.395	21	23	23.33	6.12	3.81	0.346
3	12	43.28	14.23	3.04	0.342	22	26	19.16	4.68	4.09	0.355
4	14	27.81	8.25	3.38	0.417	23	27	8.92	1.95	4.55	0.381
5	15	31.97	9.44	3.38	0.38	24	28	10.32	2.28	4.53	0.311
6	15	20.44	5.92	3.44	0.337	25	29	18.72	4.53	4.12	0.361
7	16	12.01	3.39	3.54	0.368	26	32	24.12	5.03	4.79	0.32
8	16	29.6	8.47	3.49	0.36	27	32	13.03	2.71	4.81	0.335
9	17	22.69	6.29	3.61	0.452	28	38	14.48	2.86	5.06	0.33
10	17	23.28	6.38	3.64	0.387	29	38	14.25	2.76	5.17	0.372
11	18	19.93	5.30	3.76	0.355	30	39	14.13	3.09	4.57	0.329
12	18	24.14	6.56	3.37	0.349	31	39	12.27	2.55	4.81	0.309
13	19	21.45	5.58	3.84	0.35	32	40	14.25	2.76	5.17	0.334
14	19	27.55	7.27	3.79	0.378	33	44	14.84	2.70	5.49	0.337
15	19	29.55	7.74	3.81	0.368	34	44	12.08	2.49	4.84	0.305
16	20	26.75	6.82	3.92	0.355	35	50	10.69	2.11	5.06	0.295
17	20	22.31	5.72	3.89	0.386	36	55	9.75	1.86	5.23	0.28
18	21	23.92	6.01	3.98	0.368	37	57	9.85	1.88	5.21	0.279
19	21	19.31	4.73	4.08	0.379	38	58	9.69	1.85	5.24	0.257

The sample clones used in the analyses of the main narrative are numbered 1-38 ("Clone #"; first column in each of the two parts of the table; clones #1-19 in left portion, clones #20-38 in right portion). Shown are their experimentally determined lifespans (T; second column in each table part) - ranging from 11 to almost 60 months. For each HSC, the predicted growth rate b (third column), decline rate a (fourth column) and the ratio b/a of the two values are shown (fifth column). The Hurst exponent H of the failure rate kinetic associated with each repopulation kinetic is shown in the sixth column. To facilitate clone-to-clone comparisons, all values for b and a were normalized with regard to the exponent α in the continuous model. The normalization used was $\alpha = 1 + \langle H \rangle$, where $\langle H \rangle \approx 0.346$ as determined by the methods used in the main narrative (compare Materials & Methods section).

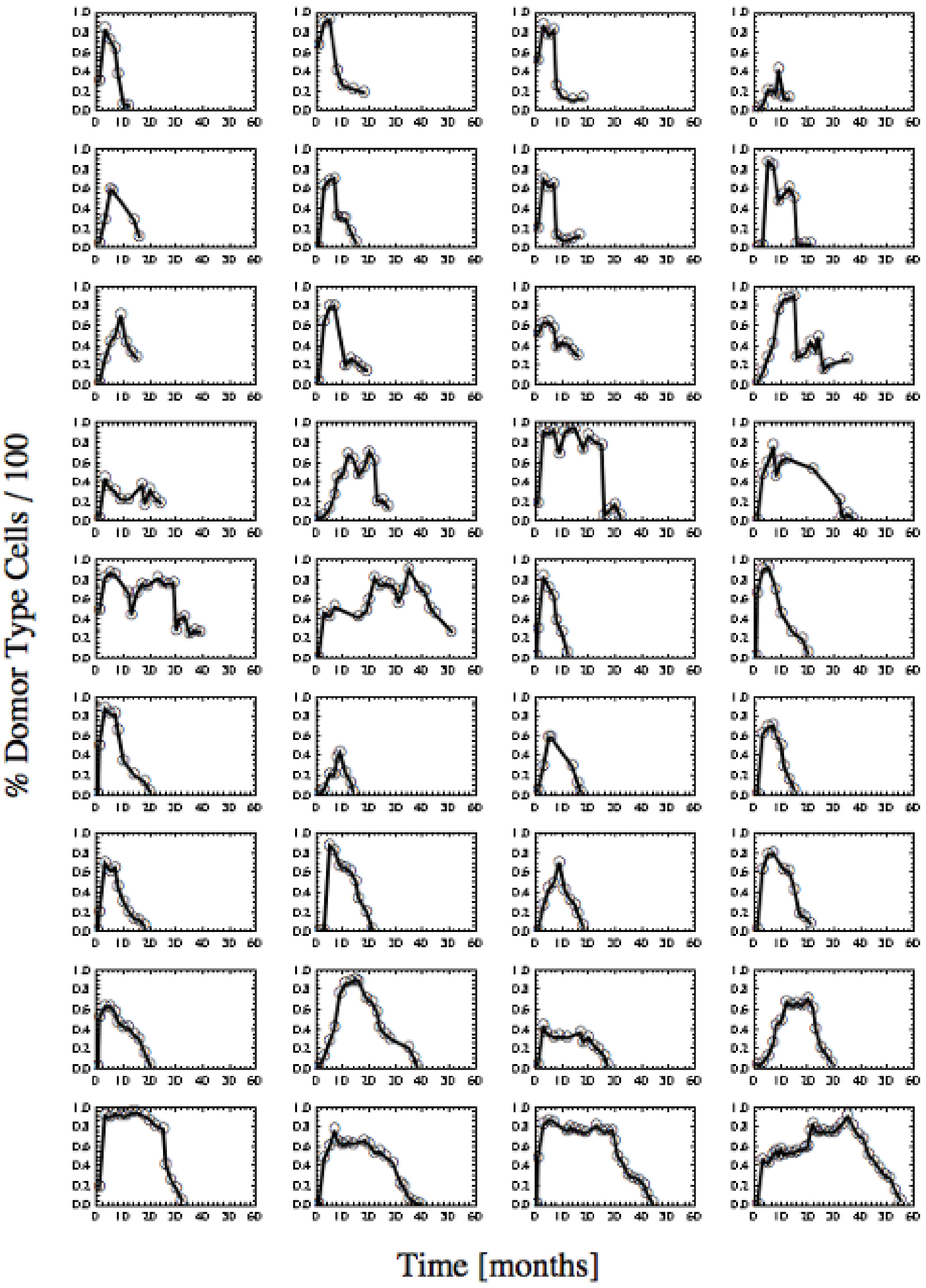

Figure S 1. Repopulation Kinetics of Clonal HSCs. Each time series shows the experimental measurements of % donor-type cells divided by 100 (vertical axis, open circles) of a single HSC transplanted and followed over time (horizontal axis) as described. To facilitate visualization of each time series shape, successive measurement points were connected (black line).

References

1. Sieburg HB, Rezner BD, Muller-Sieburg CE (2011) Predicting clonal self-renewal and extinction of hematopoietic stem cells. Proceedings of the National Academy of Sciences of the United States of America 108: 4370-5.

2. Rice J (2001) Mathematical Statistics and Data Analysis. Duxbury Press.

3. Gumbel EJ (1958) Statistics of extremes. New York,: Columbia University Press, 375 p. pp. 57010160 /L illus. 24 cm.

4. Mays L (2005) Water Resources Engineering. John Wiley & Sons, 2nd edition.
